# Metformin Targets Glucose Metabolism in Triple Negative Breast Cancer

**DOI:** 10.4172/2476-2261.1000129

**Published:** 2018-03-21

**Authors:** RS Wahdan-Alaswad, SM Edgerton, HS Salem, AD Thor

**Affiliations:** Department of Pathology, School of Medicine, University of Colorado, Anschutz Medical Campus, Aurora, USA

**Keywords:** Breast cancer, Metformin, Glucose metabolism, Triple negative breast cancer, Glycolysis

## Abstract

Metformin is the most widely administered anti-diabetic agent worldwide. In patients receiving metformin for metabolic syndrome or diabetes, it reduces the incidence and improves the survival of breast cancer (BC) patients. We have previously shown that metformin is particularly potent against triple negative breast cancer (TNBC), with a reduction of proliferation, oncogenicity and motility, inhibition of pro-oncogenic signaling pathways and induction of apoptosis. These BCs are well recognized to be highly dependent on glucose/glucosamine (metabolized through anaerobic glycolysis) and lipids, which are metabolized for the production of energy and cellular building blocks to sustain a high rate of proliferation. We have previously demonstrated that metformin inhibits lipid metabolism, specifically targeting fatty acid synthase (FASN), cholesterol biosynthesis and GM1 lipid rafts in TNBC. We also reported that glucose promotes phenotypic aggression and reduces metformin efficacy. We now show that metformin inhibits several key enzymes requisite to glucose metabolism in TNBC, providing additional insight into why metformin is especially toxic to this subtype of BC. Our data suggests that the use of metformin to target key metabolic defects in lipid and carbohydrate metabolism in cancer may be broadly applicable, especially against highly aggressive malignant cells.

## Introduction

Breast cancer (BC) morbidity and mortality remain stubbornly high worldwide, despite the fact that disease characteristics vary by geography, ethnicity, age, body mass and other factors. While the prognosis for BC patients is better in the US than in much of the world, our incidence of disease is especially high in women with obesity and type II diabetes/metabolic syndrome. Experts anticipate that in 2018, 266,000 new invasive and 64,000 new in situ breast cancer (BC) cases will be diagnosed in the US [1]. The majority of these BCs will be hormone receptor-positive (i.e. express oestrogen (ER) and/or progesterone receptor (PR)), responsive to surgery and endocrine therapy (for low risk cases), and have a good prognosis [2]. In contrast, the least common BC known as triple negative breast cancer (TNBC) lacks ER, PR and a tyrosine kinase receptor HER2, is highly aggressive and associated with the worst outcome [3–5].

Gender (being female) is the most potent risk factor for BC [6]. A family history of BC (typically involving first-degree relatives at a young age) is also an important risk factor. Heritable genetic alterations are associated with approximately one-tenth of all cases of BC [7]. Other important risk factors include age, reproductive and menstrual history, a lack of physical activity, obesity and type II diabetes [7]. For all women with both obesity and type II diabetes, the risk of BC increases by as much as 20% [8]. Less well-appreciated, gestational diabetes, pre-diabetes (the so-called metabolic syndrome) or a family history of diabetes also enhances a woman’s risk for BC [8,9]. The impact of obesity on BC risk is also influenced by age and ethnicity [10]. For example, obesity is not a strong risk factor for premenopausal Caucasian females. In older Caucasian women, however, both obesity and type II diabetes increase the risk of hormone receptor-positive BC significantly [11]. These chronic diseases are also independently associated with a worse prognosis and higher disease-associated mortality for these women [11,12]. In contrast, obesity is significantly associated with an increase in BC risk in young (premenopausal) African American (AA) women [13–15]. These women most often develop TNBC, a subtype that is usually resistant to standard chemotherapy and targeted therapeutics [11,16,17].

A historically attributed mechanism by which obesity (predominantly in a central, or abdominal distribution) promotes BC is the peripheral conversion of testosterone in adipocytes, leading to increases in circulating, bioavailable oestrogen (particularly problematic in post-menopausal women) [18]. More recent studies have shown that abdominal obesity influences BC development and outcomes through other mechanisms as well; including: systemic shifts in carbohydrate and fat metabolism, up regulation of pro-carcinogenic factors such as cytokines and growth factors (like insulin and insulin-like growth factors), modulation of the immune system and macrophage activation, as well as other systemic effects reviewed in details elsewhere [19–21]. Of note, obesity is often associated with the development of pre-diabetes (the so-called metabolic syndrome) or type II diabetes. Thus, dysregulation of carbohydrate and lipid metabolism often occurs together, typically prior to the development of BC.

With transformation of benign breast epithelial cells to the malignant phenotype, significant changes in fat metabolism and intracytoplasmic fat accumulation are often observed (particularly in TNBC). In fact, all cancer subtypes have shown an enhanced reliance on *de novo* fatty acid biosynthesis [22], irrespective of the availability of extracellular lipid derived from diet or adipose storage [23,24]. This so-called ‘lipid switch’ and the importance of Acetyl-CoA-carboxylase alpha to the malignant phenotype of BC cells have been well described by others [25,26]. We have previously shown that the anti-diabetic drug metformin has potent action against these shifts in lipid metabolism. More specifically metformin targets critical components of fatty acid synthesis [27] as well as cholesterol biosynthesis, resulting in shifts in GM1 lipid rafts and associated receptor signaling [28]. The focus of this report is to provide new data regarding the effects of metformin on carbohydrate metabolism, another critical component of malignant cell metabolism that is requisite for cancer cell survival, proliferation and progression.

## Metabolic Syndrome, Breast Cancer and Dysregulation of Carbohydrate Metabolism in Cancer

Metabolic syndrome and type II diabetes are associated with systemic dysregulation of lipid and carbohydrate metabolism. These changes disrupt a broad array of cell types and put the patient at an increased risk of cardiovascular disease and cancer [29]. Both type II diabetes and metabolic syndrome are independent risk factors for BC [30–32], although the complex mechanism by which this occurs in the breast is not well understood. The increase in serum insulin/insulin resistance and insulin-like growth factor associated with these disorders is one likely mechanism, as they are associated with an increase in breast cancer incidence and a worse prognosis [33–37]. Metabolic dysregulation is also associated with an increase in serum glucose and other energy precursors such as fructose and glucosamine that can be metabolized to adenosine triphosphate (ATP) and other factors to facilitate cancer replication and tumor growth even in a hypoxic environment [38].

Dysregulation of carbohydrate metabolism to preferentially use aerobic glycolysis is a well-recognized hallmark of cancer [39]. This metabolic reprogramming is achieved through a complex interplay of regulatory networks involving: phosphatidylinositide 3-kinase (PI3K), protein kinase B (Akt), mammalian target of rapamycin (mTOR), phosphatase and tensin homolog (PTEN), and 5′ AMP-activated protein kinase (AMPK) [40,41]. Alternative oncogenic mechanisms involving c-Myc [42], hypoxia-inducible factor 1-alpha (HIF1α) [43], epidermal growth factor receptor (EGFR), tumor protein 53 (P53) and the Met receptor have also been implicated in the transformative process by which cancer cells switch to aerobic glycolysis [44–47].

## Alterations of Glucose Metabolism in Breast Cancer

In order to meet the need for increased glucose intake from extracellular sources, cancer cells frequently upregulate membrane associated glucose transport proteins known as GLUTs [48–50], as well as associated cofactors (e.g.SGLT1) that can facilitate this process [51]. Of the various GLUT family members, GLUT1 and GLUT3 are the most highly expressed in BCs [52]. GLUT1 (SLC2A1) upregulation has been frequently reported in studies of TNBC, where it has been associated with a worse prognosis and treatment resistance [53]. In a preclinical model, GLUT1 also appears to be requisite to HER2 induced mammary tumorigenesis [54]. Other processes associated with carbohydrate metabolism have also been shown to be altered in TNBC, including oxidative phosphorylation [55] and glycolytic flux [56]. Thus, alterations of glucose metabolism are frequent, arise from multiple mechanisms and drive breast carcinogenesis (see elsewhere for a more extensive discussion [57]).

## Glucose Metabolism and Mechanisms of Metformin Action

We previously reported that glucose promotes phenotypic cancer aggression and reduces the efficacy of metformin in all molecular subtypes of BC [58]. We have shown that the mechanisms of metformin action vary by molecular subtype of the disease [58–61], and that TNBCs are especially sensitive to its anti-cancer effects. More specifically, metformin blocks cellular proliferation, reduces oncogenicity, targets stem cells, slows motility, and induces apoptosis in TNBC [58–61]. More recently we have studied the effects of metformin on mammary tumors that arise in obese vs. lean rats as well as obese overfed mice. Each of these models was used to investigate the role of metabolic dysregulation associated with obesity and carbohydrate dysregulation on mammary tumor development, progression and metformin efficacy [54,62,63]. Using these and other *in vitro* models, we seek to define mechanisms of metformin action at the subcellular level.

Metformin reduces insulin resistance, promotes glucose and lipid homeostasis, particularly in liver and skeletal muscle. The most widely recognized mechanism of metformin action is through inhibition of the mammalian target of rapamycin complex I (mTORC1) in both AMPK-dependent and independent processes [64]. Less is known about its effects on benign or malignant breast epithelial cells. We have shown that metformin attenuates a number of specific oncogenic signaling pathways in BC not widely studied in other organ systems, including: STAT3 [64], transforming growth factor-β (TGF-β) mediated activation of Smad2/Smad3 and ID1 [65], and the organic cation transporter (OCT1) [62]. We have also reported diverse targets of metformin to disrupt the aberrant lipid metabolism in BC cells. It attenuates *de novo* fatty acid synthesis through down-regulation of fatty acid synthase (FASN), via up-regulation of the microRNA 193b [27]. Metformin also inhibits 26 steps in the cholesterol synthesis pathway; resulting in a reduction of GM1 lipid raft generation and stability, as well as EGFR signaling [28].

## Metformin Attenuates Key Genes involved in Glucose Metabolism in TNBC

We have previously shown that glucose promotes BC aggression and reduces metformin efficacy *in vitro* [58]. Using a carcinogen-induced rodent model of mammary tumorigenesis, we have also reported that overfed obese animals with the equivalent of metabolic syndrome (defined by elevated serum glucose) showed a 50% increase in glucose uptake by their mammary tumor cells, associated with enhanced proliferation and metabolic “reprogramming” similar to what was observed in human BC cells *in vitro*[63].

Furthermore, we have shown that metformin had significant antitumor effects in this rodent model. These findings suggest a basis for the epidemiological and data indicating that in patients with metabolic syndrome or type II diabetes, metformin treatment reduces cancer incidence and improves survival for patients that develop the disease (in stark contrast to other anti-cancer agents including insulin that increases BC risk) [34–36]. We postulate that metformin is especially potent against TNBC because of its enhanced dependence on glucose (mediated through SLC2A1) and glutamine (mediated through SLC6A14) and its markedly aberrant mitochondrial respiration [45].

**Figure 1:**
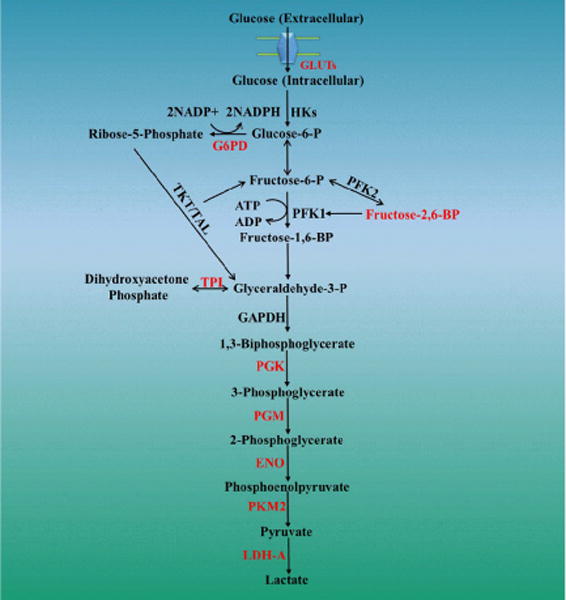
Metformin Attenuates Key Genes involved in Glucose Metabolism in TNBC.

Given that one of the primary metabolic changes observed in BC tumorigenesis is a marked increase in catabolic glucose metabolism, and metformin’s ability to disrupt these shifts, we sought to identify direct targets of the drug amongst glucose transporters and key enzymes of the glycolytic pathway. In brief, TNBC cells MDA-MB-468 and MDA-MB-231 were grown in either 17 mM or 5 mM glucose, with or without metformin. Purified mRNA was used for gene expression profiling [27, 58]. Data is shown in Figure 1. Metformin significantly down-regulated a number of glucose transporter proteins including: GLUT1 (SLC2A1), GLUT10 (SLC2A10), GLUT12 (SLC2A12), GLUT14 (SLC6A14), and Glucose-6-Phosphate transporter (SLC37A4) in MDA-MB-468 TNBC cells. Of these, GLUT1’s down-regulation by metformin is likely to have the most translational value in patients with TNBC, as it has been shown to be the predominant glucose transporter in this molecular subtype, and its expression has been associated with a worse survival. Metformin was shown to upregulate only one glucose transporter, GLUT12.

In this experiment, we also showed that metformin attenuated the expression of a number of important genes involved in glucose metabolism. Over 20 genes were shown to be down-regulated, see Table 1 and Figure 1 (metformin target genes in red). At both low and high concentrations of glucose in the culture media (data from 17mM glucose not shown but similar), metformin down-regulated G6PD and triose phosphate isomerase (TPI), a key enzyme that participates in converting glyceraldehyde-3-P to dihydroxyacetone phosphate. Additionally, metformin targeted phosphoglycerate kinase 1 (PGK), phosphoglucomutase 1 (PGM), enolase 1 (alpha) ENO, pyruvate kinase muscle 2 (PKM2), and lactate dehydrogenase A (LDHA). Of note, metformin enhanced transcription of phosphoglucomutase 5 (PGM5), phosphoglucomutase 5 pseudogene 2 (PGM5P2), aldo-keto reductase family 1 Member B10 (AKR1B10), aldo-keto reductase family 1 member C2 (AKR1C2), and pyruvate dehydrogenase kinase 4 (PDK4).

Metformin also inhibited lactose dehydrogenase (LDH). LDH functions downstream of hexokinase (HK), phosphofructokinse (PFK), and pyruvate kinase (PK). LDHA is a key enzyme that catalyzes the conversion of pyruvate to lactate. Metformin also decreased LDHA, which according to knock-down studies in BC cell lines, may reduce proliferative rates by switching cellular mitochondria to oxidative phosphorylation [66]. Additionally, metformin treatment inhibited pyruvate dehydrogenase kinase (PDK), which facilitates the conversion of pyruvate to acetyl Co-A.

We postulate that these major shifts in glucose transport and glycolysis are a major mechanism by which metformin reduces growth, oncogenesis, induces apoptosis and may enhance sensitivity to other chemotherapeutic agents. While we have little data from our lab that suggests the latter, data from others provides a rationale for this view. Reduction of LDH by genetic manipulation or the chemical inhibitor oxamate have reversed of taxol-resistance and induced apoptosis in BC cells [66].

## Material and Methods

Cell line treatment and gene profile array were previously described [27,58].

### Gene expression microarray

Microarray analysis of MDA-MB-468 cells cultured in media with 5 mM glucose in the presence or absence of 10 mM metformin. mRNA from treated cells was purified and analyzed using Affymetrix Human Gene 1.0 ST Array platform. Genes differentially down-regulated by metformin are highlighted in red. A biological triplicate experimental design was used to determine standard error.

## Statistics

Statistical considerations and calculations of metformin-mediated inhibition of cholesterol pathway genes were performed using Graph Pad Prism 7 software. Statistical analysis of the experimental data was performed using a 2-sided Student t-test. Significance was set at a P<0.05 values. Gene array samples are representative of biological replicates.

## Conclusions

Metformin is the only anti-diabetic agent with anti-cancer activity, whereas other agents used in patients with this disease or metabolic syndromes have been shown to increase cancer incidence and reduce cancer-associated survival. Metformin has broad effects on multiple targets of the dysregulated lipid and carbohydrate metabolism associated with BC, and more specifically, TNBC. In this report, we demonstrate that metformin specifically reduces the expression of key glucose transporters in TNBC, including GLUT1. We also show that it reduces transcription of key enzymes in the glycolytic pathway that are critical for cancer replication and survival. Because of the increasing prevalence of obesity, the relationships between excess body weight and cancer development and the underlying biological mechanisms need to be further investigated to prevent and treat BC in the future.

## Figures and Tables

**Table 1:** Metformin attenuated the expression of a number of important genes involved in glucose metabolism. Over 20 genes were shown to be down-regulated.

Gene Assignment	Gene Symbol	Ref Seq	Fold-Change (G5 Met vs. G5)	P-Value
**GLUCOSE TRANSPORTERS**				
Solute Carrier Family 2 Member 1 (Facilitated Glucose Transporter)	SLC2A1	NM_006516	−1.71564	1.88E-05
Solute Carrier Family 2 Member 10 (Facilitated Glucose Transporter)	SLC2A10	NM_030777	−1.8308	0.000166115
Solute Carrier Family 6 Member 14 (Amino Acid Transporter)	SLC6A14	NM_007231	−2.44598	2.17E-06
Solute Carrier Family 37 Member 4 (Glucose-6-Phosphate Transporter)	SLC37A4	NM_001164277	−2.38101	4.34E-06
Solute Carrier Family 2 Member 12 (Facilitated Glucose Transporter)	SLC2A12	NM_145176	1.5	4.25E-05
**GLUCOSE METABOLISM**				
**Glycolysis**				
Enolase 1 (alpha)	ENO1	NM_001428	−1.5703	7.57E-07
Glucose-6-Phosphate Isomerase	GPI	NM_000175	−1.64235	1.76E-05
Phosphofructokinase, Liver	PFKL	NR_024108	−1.51114	2.73E-05
Phosphoglycerate Kinase 1	PGK1	NM_000291	−2.49786	7.32E-08
Phosphoglucomutase 1	PGM1	NM_002633	−1.55441	6.65E-05
Triosephosphate Isomerase 1	TPI1	NM_000365	−1.52868	0.001659
Glucose-6-Phosphatase Catalytic Subunit 3	G6PC3	NM_138387	−2.05414	6.25E-05
6-Phosphofructo-2-Kinase/Fructose-2,6-Biphosphatase 4	PFKFB4	NM_004567	−2.2822	1.12E-06
Transketolase	TKT	NM_001135055	1.52762	3.90E-05
Pyruvate Kinase, Muscle	PKM2	NM_182470	−1.72714	4.15E-06
Lactate Dehydrogenase A	LDHA	NM_005566	−1.86317	1.22E-08
Phosphoglucomutase 5	PGM5	NM_021965	1.51945	0.001669
Phosphoglucomutase 5 Pseudogene 2	PGM5P2	NR_002836	1.7348	0.000269
Aldo-Keto Reductase Family 1 Member B10	AKR1B10	NM_020299	2.9025	1.08E-06
Aldo-Keto Reductase Family 1 Member C2	AKR1C2	NM_001354	2.63487	1.44E-06
Pyruvate Dehydrogenase Kinase 3	PDK3	NM_005391	−2.41605	1.79E-05
Pyruvate Dehydrogenase Kinase 4	PDK4	NM_002612	4.13308	2.07E-06
Pyruvate Dehyrogenase Phosphatase Catalytic Subunit 1	PDP1	NM_001161778	2.83315	5.78E-06
Prenyl (Decaprenyl) Diphosphate Synthase Subunit 1	PDSS1	NM_014317	−2.09161	8.58E-05
Phosphoglycerate Mutase 1 (Brain)	PGAM1	NM_002629	−2.54865	5.42E-07
Phosphoglycerate Mutase Family Member 4	PGAM4	NM_001029891	−2.26653	2.89E-06
UDP-Glucose 6-Dehydrogenase	UGDH	NM_003359	−2.0062	3.93E-05
**Gluconeogenesis**				
Pyruvate Carboxylase	PC	NM_001040716	−2.39828	7.29E-06
**Tricarboxylic Acid Cycle (TCA)**				
ATP Citrate Lyase	ACLY	NM_001096	−4.25837	2.97E-10
Aconitase 1	ACO1	NM_002197	−1.56798	0.001286
Dihydrolipoamide S-Acetyltransferase	DLAT	NM_001931	−1.76732	1.98E-07
Isocitrate Dehydrogenase 2 (NADP+)	IDH2	NM_002168	−1.75573	6.09E-06
Oxoglutarate (Alpha-Ketoglutarate) Dehydrogenase (Lipoamide)	OGDH	NM_002541	−1.78156	0.000117
Pyruvate Dehydrogenase (Lipoamide)	PDHA1	NM_000284	−1.61653	1.82E-05
Succinate Dehydrogenase Complex Subunit A (Flavoprotein)	SDHA	NM_004168	−1.64205	2.66E-05
Succinate Dehydrogenase Complex Subunit C (Integral Membrane Protein)	SDHC	NM_003001	−1.59586	5.46E-05
